# Analysis of Serum Paraoxonase 1 Using Mass Spectrometry and Lectin Immunoassay in Patients With Alpha-Fetoprotein Negative Hepatocellular Carcinoma

**DOI:** 10.3389/fonc.2021.651421

**Published:** 2021-04-06

**Authors:** Xinyi Cao, Zhao Cao, Yuyin Shao, Chao Liu, Guoquan Yan, Xinmin Meng, Lei Zhang, Chen Chen, Guiyue Huang, Hong Shu, Haojie Lu

**Affiliations:** ^1^ Institutes of Biomedical Sciences, Fudan University, Shanghai, China; ^2^ Department of Clinical Laboratory, First Affiliated Hospital of Guangxi Medical University, Nanning, China; ^3^ Beijing Advanced Innovation Center for Precision Medicine, Beihang University, Beijing, China; ^4^ Department of Clinical Laboratory, Cancer Hospital of Guangxi Medical University, Nanning, China; ^5^ Department of Chemistry, Fudan University, Shanghai, China; ^6^ NHC Key Laboratory of Glycoconjugates Research, Fudan University, Shanghai, China

**Keywords:** alpha-fetoprotein-negative hepatocellular carcinoma, PON1, biomarker, glycoproteomics, mass spectrometry, lectin ELISA

## Abstract

The diagnosis of AFP (alpha-fetoprotein)-negative HCC (hepatocellular carcinoma) mostly relies on imaging and pathological examinations, and it lacks valuable and practical markers. Protein N-glycosylation is a crucial post-translation modifying process related to many biological functions in an organism. Alteration of N-glycosylation correlates with inflammatory diseases and infectious diseases including hepatocellular carcinoma. Here, serum N-linked intact glycopeptides with molecular weight (MW) of 40–55 kDa were analyzed in a discovery set (n = 40) including AFP-negative HCC and liver cirrhosis (LC) patients using label-free quantification methodology. Quantitative lens culinaris agglutin (LCA) ELISA was further used to confirm the difference of glycosylation on serum PON1 in liver diseases (n = 56). Then, the alteration of site-specific intact N-glycopeptides of PON1 was comprehensively assessed by using Immunoprecipitation (IP) and mass spectrometry based ^16^O/^18^O C-terminal labeling quantification method to distinguish AFP-negative HCC from LC patients in a validation set (n = 64). Totally 195 glycopeptides were identified using a dedicated search engine pGlyco. Among them, glycopeptides from APOH, HPT/HPTR, and PON1 were significantly changed in AFP-negative HCC as compared to LC. In addition, the reactivity of PON1 with LCA in HCC patients with negative AFP was significantly elevated than that in cirrhosis patients. The two glycopeptides HAN^253^WTLTPLK (H5N4S2) and (H5N4S1) corresponding to PON1 were significantly increased in AFP-negative HCC patients, as compared with LC patients. Variations in PON1 glycosylation may be associated with AFP-negative HCC and might be helpful to serve as potential glycomic-based biomarkers to distinguish AFP-negative HCC from cirrhosis.

**Graphical Abstract:**
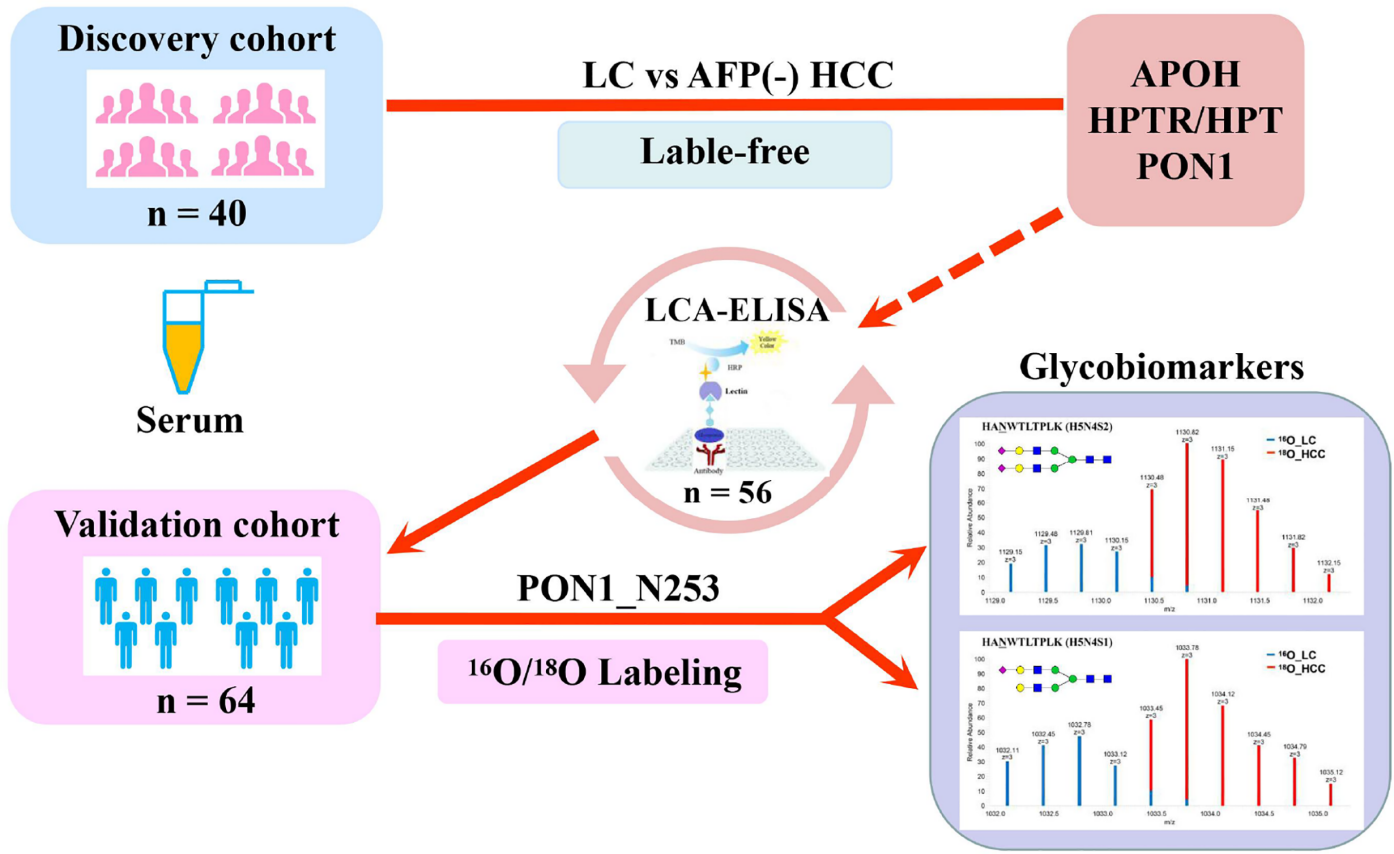


## Introduction

As the most common human cancer, hepatocellular carcinoma (HCC) is a dominant reason for cancer-related death all around the world (1, 2). China is among the countries with the most serious liver cancer epidemic (3). Most cases of liver cancer in clinical practice develop on the background of hepatic cirrhosis (4). Continuous screening for HCC progression could help to increase survival in cirrhosis patients (5). Therefore, monitoring the development of liver cancer is a key issue for the management of cirrhosis patients.

Alpha-fetoprotein (AFP) is controversial for its clinical application in the clinical detection of liver cancer. Serum AFP is a widespread diagnostic biomarker for HCC, however, with limited diagnostic power (6–8). The sensitivity of ultrasound combined with AFP is only approximately 60% (9). In addition, about one-third of HCC patients have low or normal AFP concentration in the serum, and the diagnosis of HCC in this condition is a tough challenge (10). Several studies are in progress to discover novel biomarkers in HCC, especially for AFP-negative patients.

As an important and ubiquitous posttranslational modification, protein glycosylation plays an important role in pathological and physiological processes (11, 12). In humans, nearly 70% of proteins are glycosylated (13). Moreover, N-glycosylation regulates the correct folding, transport, and biological function of various proteins (14) and has been shown to be involved in cancer, inflammatory diseases, and autoimmune diseases by participating in proliferation, cell–matrix interactions, migration, and differentiation (15, 16). Glycosylation has been reported to be associated with most cancer serum biomarkers (17). For example, a serum glycomic-based approach can be applied to evaluate the risk for the development of HCC in patients with compensated cirrhosis (18). In recent years, changes in the glycosylation of liver disease-related sera proteins have attracted wide attention. For instance, the researchers employed MALDI-TOF mass spectrometry systems to profile glycan of serum AGP (19). It has been reported that the trifucosylated tetra-antennary glycan structure of AGP could distinguish HCC from cirrhosis. There are increasing pieces of evidence that glycosylation changes of serum proteins are closely related to cancer progression (20–22). Unique alterations of tumor-associated N-glycosylation have several inherent advantages, such as providing distinct biomarkers indicating significant and amplified changes (23). Characterizing the heterogeneity and dynamism of glycosylation will help to better understand the molecular pathogenesis of cancer and shed light on new clues for diagnosis, prognosis, and treatment (24, 25).

The relevance between human diseases and glycosylation has attracted researchers’ attention. The specific differences of plasma protein glycosylation between liver cirrhosis and liver cancer have been revealed by mass spectrometry analysis (26–28). It was uncovered that about 24 kinds of glycoproteins were the main glycoproteins in plasma, of which the 40-kDa band (40–55 kDa) accounts for more than half (29). In our previous study, N-glycopeptides of 40–55 kDa band in LC and HCC were quantified. TPLTAN^205^ITK (H5N4S2F1) and (H5N5S1F1) of IgA_2_ were changed significantly during the development of HCC (22).

In the present study, we aimed to discover new diagnostic biomarkers to distinguish AFP-negative HCC from LC patients based on glycoproteomic technology and confirmed the differential expression of the identified glycopeptides in a validation set.

## Materials And Methods

### Subjects Samples

Samples were collected from Guangxi Medical University Cancer Hospital, including 80 cases of cirrhosis and 80 cases of liver cancer (**Table 1**). Medical Ethics Committee of Guangxi Medical University Cancer Hospital approved the acquisition and use of these specimens. All patients gave informed consent. Cirrhotic patients were collected retrospectively, and those who had received local treatment or incomplete clinical data were excluded. Cirrhosis was confirmed by B-ultrasound examination in the presence of chronic liver disease. Hepatocellular carcinoma diagnosis was based on CT, MRI characteristics, or the results of pathology examination.

**Table 1:** General information and clinical characteristics of the participants.

	Liver cirrhosis	AFP-negative HCC
	n = 80^*^	n = 80^*^
Age	48 (20–67)	48 (20–79)
Gender (M:F)	63:17	72:8
AFP (ng/ml)	4.1 (0.8–19.9)	6.3 (0.4–19.0)
ALT (U/L)^a^	28.8 (6–86)	86.3 (13–601)
AST (U/L)^a^	37.1 (18–105)	90.0 (19–884)
HBsAg^+^ (%)	87.5	91.3

The serum AFP <20 ng/ml was considered to be AFP negative.

^*^Eighty patients in each group are composed of a discovery set (n = 20), samples for ELISA (n = 28) and a validation set (n = 32).

a ALT and AST levels were determined by ARCHITECT c16000 (Abbott, Sligo, Ireland).

### Sample Preparation

For the discovery phase, the serum specimens were obtained from 20 patients with liver cirrhosis (four biological repetitions, each pooled from five random individuals) and 20 patients with AFP-negative hepatocellular carcinoma (four biological repetitions, each pooled from five random individuals). Serum AFP level <20 ng/ml was considered to be AFP negative, using standard kits (Abbott Labs). In addition, traditional lectin ELISA was conducted for each patient of another 56 patients for further confirmation. For the validation phase, an independent cohort including 32 patients with LC and 32 with AFP-negative HCC were assessed individually by ^16^O/^18^O C-terminal labeling quantitation method after IP, which were entirely separated from discovery set samples. Serum samples were stored at −80°C and handled identically. The clinical characteristics of LC and HCC patients were shown in **Table 1**. The study excluded patients with other virus infection or autoimmune diseases.

### Digestion of Proteins and Glycopeptide Enrichment

20 µg of haptoglobin (Hp) (Calbiochem, San Diego, USA) and 5 µl serum were separated by SDS-PAGE (10%). Coomassie blue was used to visualize the protein bands. The *β* chain (40 kDa) of Hp and the 40-kDa band of serum lane were excised. Subsequently gel pieces were reduced and alkylated. Trypsin (Promega, Madison) was added at the temperature of 37°C overnight. Then peptides were obtained and lyophilized. The Glycopeptide Enrichment Kit was used according to the instructions (Novagen, Darmstadt, Germany).

### Mass Spectrometry Analysis

An EASY-nano-LC 1000 system (Thermo Scientific, Waltham, MA) with an Orbitrap Fusion mass spectrometer (Thermo Scientific, San Jose, CA) was used. The mixture was loaded onto the trap column (2 cm × 100 µm, PepMap C18) and the analytical column (25 cm × 75 µm, PepMap C18) by using a gradient of 1 to 25% solvent B in 60 min, followed by an increase to 45% B in 20 min. Parameters used for MS data acquisition were as described (22). The intact glycopeptides were identified by pGlyco (http://pfind.ict.ac.cn/software/pGlyco/index.html) (Version 2020.05.27) and quantified by pQuant (22, 30).

### Immunoprecipitation

Serum was diluted with lysis buffer (Beyotime, Shanghai, China). In this procedure, 20 µl of protein G agarose slurry (Roche, Basel, Switzerland) was added to the diluted serum at 4°C for 3–4 h incubation to remove IgG interference. After high speed centrifugation, 30 µl of protein G agarose slurry (Roche) and anti-PON1 antibody (ab92466, Abcam, UK) were sequentially added to the IgG-depleted serum for overnight incubation. Then the beads were washed with the same lysis buffer for five times. IP samples were handled with SDS-PAGE for in-gel digestion and glycopeptide enrichment.

### ^16^O/^18^O Labeling of Glycopeptides

Immobilized trypsin (Thermo Scientific, Rockford, IL) was added and dried with a vacuum centrifuge. H_2_^16^O/H_2_^18^O (97%, Cambridge Isotope Laboratories, Andover, MA) was added respectively for 24 h at the temperature of 37°C. ^16^O- and ^18^O-labeled digests were pooled by equal volumes before mass spectrometry analysis. The ^16^O/^18^O labeled N-glycopeptides were identified and quantified according to a previous study (22).

### Protein ELISA and Lectin-ELISA Assay

Levels of PON1 in serum were determined with ELISA kits (R&D, Minneapolis, USA). Samples were diluted 1:50 in phosphate-buffered saline before lectin-ELISA. The 96-well plate was coated with diluted capture antibody. After overnight incubation, each well was blocked with (3%) BSA in PBS for more than 2 h. Then, the coated antibody was reacted with the oxidation buffer (50 mM citric acid, 100 mM NaIO_4_) for 1 h without light. After washing, each well of plate was applied with 100 µl diluted serum. After incubation, each well was washed with PBST (0.05% Tween20 in PBS), and 1 µg/ml biotinylated LCA (Vector, Burlingame, CA) was then added at room temperature for 2 h. HRP-streptavidin was added to the plate followed by TMB and stop solution. OD value at 450 nm was measured. Twenty-eight LC and 28 AFP-negative HCC patients were conducted individually. ELISA Index was defined to be OD value of PON1 bound to lectin LCA, divided by OD value of protein PON1.

### Statistical Analysis

Figures were plotted with GraphPad Prism (version 8.0, GraphPad Software Inc.) and OriginPro 8 software. Statistical comparisons were calculated using t-test. P <0.05 was considered to be statistically significant. All tests for significance were two-tailed.

## Results

### Reproducibility of Label-Free Quantitation

**Figure 1:**
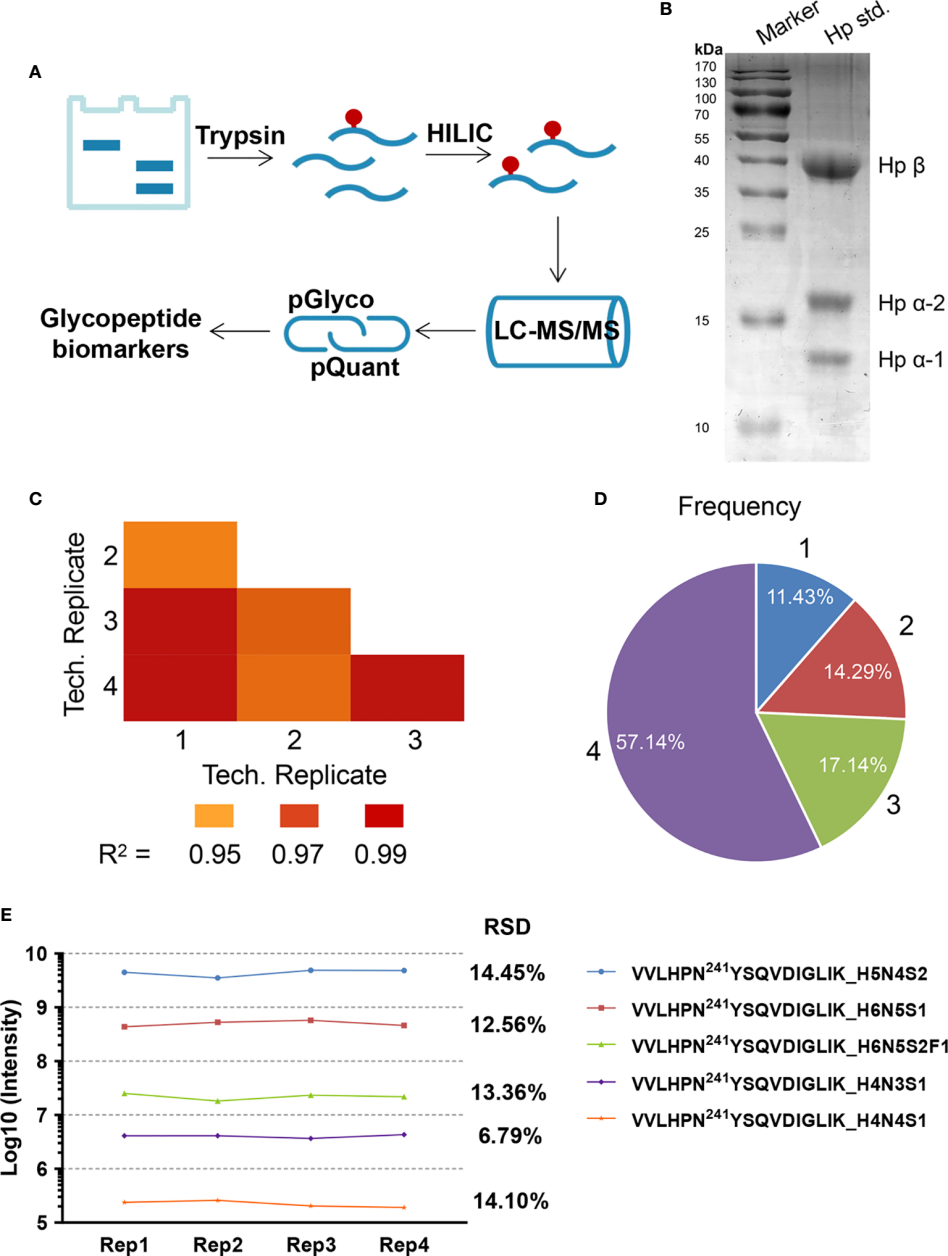
**(A)** Workflow for label-free quantification of N-glycopeptides. **(B)** Standard haptoglobin (Hp) was separated by 1D gel. **(C–E)** Reproducibility analyses of four technical replicates performed at four different times. **(C)** The Pearson Correlation Coefficient R^2^ values in technical repetition. **(D)** The frequency of N-glycopeptide identification in technical repetition. **(E)** The reproducibility of glycopeptide intensity at N241 site in the four technical repeats.

Hp was utilized as standard glycoprotein to assess the stability of label-free quantitation methodology (**Figure 1A**). After running for each serum experiment, the blank was run in order to ensure no carry-over. A representative gel image was shown in **Figure 1B**. After enrichment, N-glycopeptides of standard Hp were investigated by nano LC-HCD-MS/MS. We further selected the high-abundance glycosites N184 and N241 to evaluate the quantitative reproducibility through four independent replicates at four different times. In order to simplify the glycan annotation, a four-digit nomenclature in HNSF order was used (H, Hexose; N, N-acetylhexosamine; S, Sialic acid; F, Fucose). As described in the figure (**Figure 1C**), the Pearson Correlation Coefficient R^2^ values of the four technical repeats ranged from 0.955 to 0.998, indicating fine reproducibility. Pie chart (**Figure 1D**) showed the frequency of intact glycopeptide quantification in four replicates, in three, in two, or only in one. The results indicated that 88.57% of glycopeptides were identified with at least two repeats. The quantification reproducibility of N241 glycopeptides was also carried out, and the difference of abundance was five orders of magnitude (**Figure 1E**). At site 241, the RSD of the most abundant glycopeptide (VVLHPN^241^YSQVDIGLIK_H5N4S2) was 14.45%; the low abundant glycopeptide of VVLHPN^241^YSQVDIGLIK_H4N4S1 was 14.10% showing a good reproducibility of the method.

### Quantification for N-Glycopeptides of 40-kDa Band

**Figure 2:**
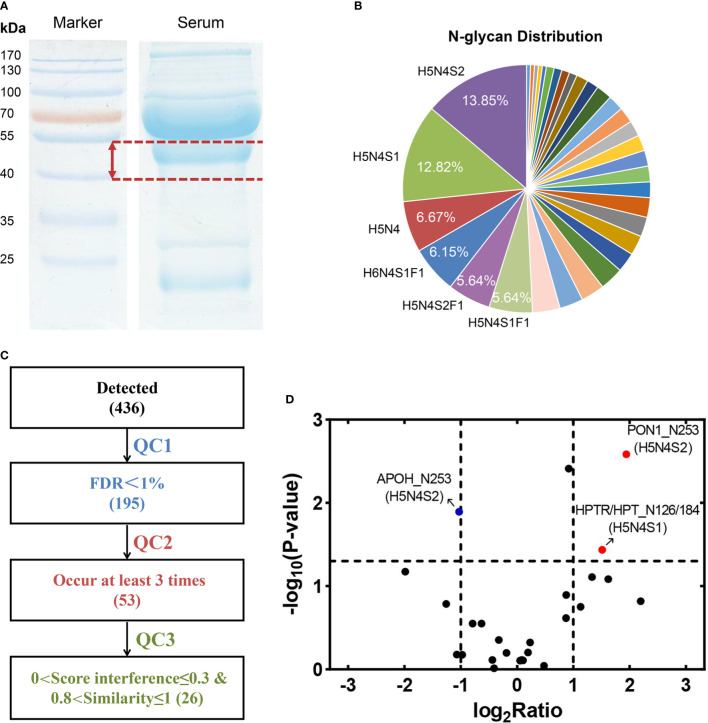
**(A)** Equal volumes of serum were acquired to separate 40-kDa band. **(B)** A total of 195 N-linked glycopeptides were identified, belonging to 33 kinds of glycan compositions. **(C)** Based on four biological repeats (each pooled from five randomly selected individuals), 26 N-linked glycopeptides appeared more than three times (QC2) and passed strict screening criteria (QC3) were considered. **(D)** Three glycopeptides corresponding to APOH, PON1, and HPTR/HPT were changed significantly in AFP-negative HCC compared with LC patients.

**Figure 3:**
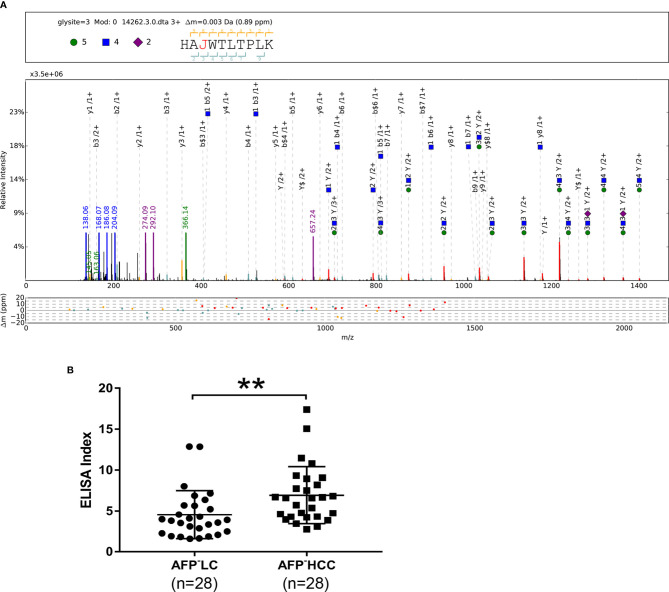
**(A)** pGlyco annotations of HAN^253^WTLTPLK (H5N4S2) of PON1. “J” represents the glycosylation site “N”; green circle: H, hexose; blue square: N, N-acetylglucosamine; purple rhombus: S, sialic acid. The upper frame of each spectrum is designed to annotate peptide sequence and glycan composition. The mass deviations of the annotated peaks are shown in the box below. **(B)** Scatter plot of ELISA Indices (LC, n = 28; HCC, n = 28). **p < 0.01.

The label-free glycopeptide quantification method was performed to obtain comparisons of AFP-negative HCC and LC. Equal amounts of AFP-negative HCC and LC sera (one pool of five individuals) were obtained to separate target bands (**Figure 2A**). In total, 195 glycopeptides were identified (**Supplementary Table S1**) and corresponded to 33 kinds of glycan compositions. For instance, H5N4S2 was considered as the most common glycan composition in the experiment (**Figure 2B**). According to the four biological repeats, 53 N-glycopeptides appeared at least three times (**Figure 2C**). Among them, 26 N-glycopeptides passed the strict filtering criteria (0.8<similarity ≤ 1 and 0<score interference ≤ 0.3) (**Supplementary Table S2**). As the cutoff criterion was set to fold change (FC) >2 or <0.5, three differentially expressed glycopeptides were identified in AFP-negative HCC samples compared to LC samples, including APOH_N253 (H5N4S2), PON1_N253 (H5N4S2), and HPTR/HPT_N126/184 (H5N4S1) (**Figure 2D**). Compared with LC patients, PON1_N253 (H5N4S2) and HPTR/HPT_N126/184 (H5N4S1) were significantly up-regulated in AFP-negative HCC (p = 0.0026 and p = 0.0366, respectively), while APOH_N253 (H5N4S2) was considerably decreased (p = 0.0127). Interestingly, the protein level of PON1 was shown to be down-regulated in AFP-negative HCC in our recent study (31), which is opposite to the variation in glycopeptide abundance. Moreover, the increase of fucosylation and sialylation in patients’ sera could play an essential role in the development of HCC (32). Finally, we selected PON1 as target glycoprotein in our study for further confirmation, whose glycosylation levels in AFP-negative HCC had only a few studies (33). Representative pGlyco annotation of PON1_N253 (H5N4S2) was shown in **Figure 3A**.

### Measurement of PON1 Glycosylation Using ELISA Index

It was reported that LCA could recognize core fucosylated bi-antennary or tri-antennary N-glycans (34). Therefore, LCA was chosen as the lectin for pre-clinical assessment of glycan alterations of PON1. Serum was 50-fold and 400-fold diluted for LCA-ELISA and protein ELISA, respectively. Twenty-eight LC samples and 28 AFP-negative HCC samples were used to determine the ELISA Index. Concentrations of PON1 in AFP-negative HCC patients (231.21 ng/ml; range 102.26–285.97 ng/ml) were lower than those in LC patients (331.38 ng/ml; range 135.82–575.79 ng/ml). ELISA Indices of PON1 in AFP-negative HCC patients (6.93; range 2.74–17.39) were significantly higher than LC patients (4.54; range 1.58–12.87) (**Figure 3B**).

### Relative Quantitation of Site-Specific N-Glycans on PON1

**Figure 4:**
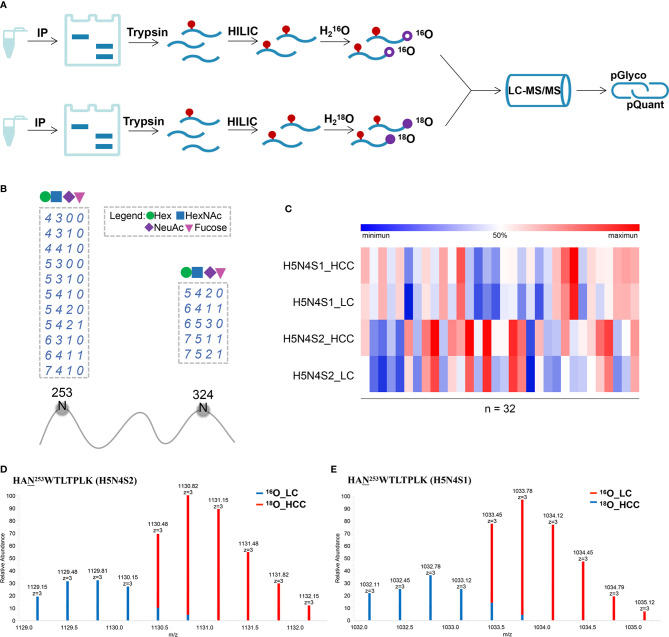
**(A)** Workflow for labeling quantification of N-glycopeptides. **(B)** Visualization of all identified glycosylation sites (N) on PON1. The glycan compositions are expressed by the numbers of Hex, HexNAc, NeuAc, and Fucose (dashed box). **(C)** Heat map of the two differentially expressed intact glycopeptides in PON1 (site N253) between AFP-negative HCC and cirrhosis (LC, n = 32; HCC, n = 32). **(D)** MS^1^ spectrum of HAN^253^WTLTPLK (H5N4S2). pQuant reported that the ^18^O/^16^O ratio was 2.82. **(E)** MS^1^ spectrum of HAN^253^WTLTPLK (H5N4S1). pQuant reported that the ^18^O/^16^O ratio was 2.61.

To further discover and confirm N-glycosylation of PON1 in AFP-negative HCC, ^16^O/^18^O-based labeling method was applied (22, 35). A total of 32 AFP-negative HCC and 32 LC patients were used for relative quantification in individual patients. Serum PON1 was immune precipitated from equal volume of serum and separated by SDS-PAGE. After in-gel digestion and glycopeptide enrichment, the peptide mixtures were labeled by H_2_^16^O/H_2_^18^O and subjected to LC-MS/MS analysis (**Figure 4A**). The predicted N-glycosylation sites in PON1 were also considered (http://www.cbs.dtu.dk/services/NetNGlyc/). Considering N253 and N324 were the most informative sites in PON1 (36, 37), the glycosylation heterogeneity of these two sites was further studied. 16 glycoforms of the two glycosites (N253 and N324, detailed information shown in **Supplementary Table S3**) on PON1 were identified by pGlyco (FDR < 1%, **Figure 4B**). Among the identified glycopeptides, the intensity of HAN^253^WTLTPLK (H5N4S2) and (H5N4S1) was significantly increased in AFP-negative HCC patients (**Figure 4C**, detailed information shown in **Supplementary Table S4**), which was partially consistent with the result of label-free. Representative MS^1^ spectra and pGlyco annotations of HAN^253^WTLTPLK (H5N4S2) and (H5N4S1) were shown in **Figures 4D, E** and **Supplementary Figure S1**. Thus, HAN^253^WTLTPLK (H5N4S2) and (H5N4S1) may be useful in distinguishing AFP-negative HCC from cirrhosis.

### Discussion

Currently, there are many advances in the treatment of liver cancer, including liver transplantation, surgical resection, and interventional therapy (38, 39). However, numerous HCC patients are diagnosed only after the obvious clinical symptoms appear in the middle or late stage, which makes early diagnosis a top priority (40, 41). At present, AFP is used as a serum biomarker to assist in the diagnosis or screening of high-risk liver cancer patients (42), but approximately 30–40% of overall hepatocellular carcinoma patients have very low serum AFP levels (<20 ng/ml), which is called AFP-negative HCC (43). The diagnostic rate of AFP-negative hepatocellular carcinoma is only 10.4% (43). Recently, Wang et al. have summarized the novel biomarkers with potential value in the diagnosis of AFP-negative HCC. Numerous glycoproteins have been confirmed to screen AFP-negative liver cancer in clinical studies (44), including Golgi protein 73 (GP73) (45), dickkopf-1 (DKK1) (46), angiopoietin-like protein 2 (ANGPTL2) (47), and haptoglobin (Hp) (48). While few of them have good clinical application value, most of them have not been studied specifically for the diagnosis of AFP-negative hepatocellular carcinoma. Therefore, for AFP-negative HCC patients, there is an urgent need for new, economical, and effective biomarkers to predict the diagnosis of HCC.

Glycosylation of serum glycoproteins is always associated with tumor progression. Changes in protein glycosylation are becoming increasingly popular to be used as diagnostic, prognostic, and therapeutic drug target indicators (49). Most of the N-glycans found in the whole serum are attached to the serum proteins produced in the liver. Changes in glycosylation of specific serum proteins secreted by the liver, such as haptoglobin, hemopexin, and vitronectin have been associated with HCC (50). In the present research, the N-glycopeptides of serum glycoprotein cluster with molecular weight of 40–55 kDa were quantified in LC *versus* AFP-negative HCC patients. Three differentially expressed intact N-glycopeptides belonging to APOH, PON1, and HPTR/HPT were screened. PON1_N253 (H5N4S2) and HPTR/HPT_N126/184 (H5N4S1) were up-regulated in AFP-negative HCC samples in this study. Whereas, APOH_N253 (H5N4S2) was reduced in HCC compared with LC patients. Then, the ELISA Indexes of protein ELISA and LCA-ELISA were used to validate glycan alterations of serum PON1 in AFP-negative patients with HCC. It was revealed that the changes of glycans on PON1 were significant.

PON1 is a high-density lipoprotein (HDL)-related serum enzyme that is mainly synthesized in the liver and released into the blood stream (51), which plays an important role in the prevention of vascular disease by metabolizing oxidized lipids (52). It has been found that changes in circulating PON1 levels are associated with various diseases involving oxidative stress reaction and inflammation (53). In our latest study, protein levels of PON1 in LC patients were elevated significantly than those in AFP-negative HCC patients (31), which is consistent with our PON1 ELISA results in this study. While this seemingly opposite trend may imply a dramatic change in PON1 glycosylation, which is independent of protein level. The fucosylation of PON1 was reported in HCC and lung cancer (33, 54), and there were two informative N-glycosylation sites (N253 and N324) on PON1 (36, 37, 55). A variety of studies have confirmed changes in the N-glycosylation state of PON1, while only a few studies have proved the site-specific glycosylation of PON1 using glycoproteomic approach.

Intact glycopeptide analysis including glycosylation site and site-specific glycan is crucial for the understanding of the biological significance of glycosylation (56). Based on labeling quantification, two N-glycopeptides HAN^253^WTLTPLK (H5N4S2) and (H5N4S1) of PON1 with high abundance have been realized to be increased significantly in AFP-negative HCC as compared with LC patients. It is the first reported study about site-specific PON1 glycosylation in AFP-negative liver disease conditions. However, our present study has limitations in terms of sample size and the absence of controls with benign liver diseases. This study has clarified the existence of potential biomarkers, such as the two glycopeptides of PON1 in AFP-negative liver cancer. Other candidate biomarkers should be further studied using other proteomic methodology.

To sum up, MS-based quantification methods combining labeling/label-free and lectin-based ELISA were used to screen and verify glycopeptide biomarkers in patients with LC and AFP-negative HCC. The results showed that two site-specific glycoforms on PON1 were significantly elevated in AFP-negative HCC and could be potential biomarkers to screen of AFP-negative HCC *versus* LC patients. For the clinical application of new-found biomarkers, preclinical validation is usually required with high-throughput quantitative methods and independent large sample sets (57). Multicenter studies on large samples are needed to evaluate the practicability of the results and are expected to translate them into clinical practice.

## Conclusion

In conclusion, we found that N-glycosylation of PON1 played a key role in AFP-negative HCC tumorigenesis. Moreover, HAN^253^WTLTPLK (H5N4S2) and (H5N4S1) of PON1 could be served as potential diagnostic indicators for distinguishing AFP-negative HCC from cirrhosis.

## Data Availability Statement

The proteomics data and analyzed result datasets have been partially deposited to the ProteomeXchange Consortium (http://proteomecentral.proteomexchange.org) via its official member iProX repository (58) with the dataset identifier PXD023500.

## Ethics Statement

The studies involving human participants were reviewed and approved by the Research Ethics Committee of Cancer Hospital of Guangxi Medical University (no. LW2019043). The patients/participants provided their written informed consent to participate in this study.

## Author Contributions

HL and HS conceived and designed the research. All authors were responsible for acquisition and analysis of data. Furthermore, XC, YS, and XM performed the research. CL, GY, and LZ provided technical assistance. XC, ZC, CC, and GH performed the data analyses. HS and XC drafted the manuscript. HS and HL revised and commented on the draft. All authors contributed to the article and approved the submitted version.

## Funding

This work was supported by the National Key Research and NSF of China (Grants 21974025), Development Program of China (2016YFA0501303), Natural Scientific Foundation for the Youths of Guangxi province of China (2015GXNSFBA139175), Natural Science Fund Project of Guangxi Province of China (2018GXNSFAA281053) and the Self-raised Scientific Research Fund of the Ministry of Health of Guangxi Province (Z2015587).

## Conflict of Interest

The authors declare that the research was conducted in the absence of any commercial or financial relationships that could be construed as a potential conflict of interest.

The handling editor declared a shared affiliation, though no other collaboration, with one of the authors with several of the authors ZC, CC, GH, XM, HS.

## Abbreviations

AST, Aspartate amino transferase; AFP, Alpha-fetoprotein; ALT, Alanine aminotransferase; ACN, Acetonitrile; AGP, Alpha-1 acid glycoprotein; ANGPTL2, Angiopoietin-like protein 2; BSA, Bovine serum albumin; ELISA, Enzyme linked immunosorbent assay; DKK1, Dickkopf-1; F, Fucose; FA, Formic acid; GP73, Golgi protein 73; H, Hexose; HCD, Higher energy collisional dissociation; HBsAg, Hepatitis B surface antigen; HCC, Hepatocellular carcinoma; HDL, High-density lipoprotein; Hp, Haptoglobin; HRP, Horseradish peroxidase; IAA, Iodoacetamide; IP, Immunoprecipitation; LC, Liver cirrhosis; MW, Molecular weight; LCA, Lens culinaris agglutin; LC-MS, Liquid chromatography mass spectrometry; MRI, Magnetic resonance imaging; N, N-acetylhexosamine; PBS, Phosphate buffered saline; PON1, Serum paraoxonase/arylesterase 1; RSD, Relative standard deviation; S, Sialic acid; TMB, 3,3′,5,5′-Tetramethylbenzidine; TFA, Trifluoroacetic acid; HILIC, Hydrophilic interaction liquid chromatography.
