# Supplementary material for: Analysis of Serum Paraoxonase 1 Using Mass Spectrometry and Lectin Immunoassay in Patients With Alpha-Fetoprotein Negative Hepatocellular Carcinoma
Source: Front Oncol. 2021 Apr 6;11:651421. doi: 10.3389/fonc.2021.651421 (PMC8056865; doi:10.3389/fonc.2021.651421)
Supplement: Supplementary file 2 [file Table_2.doc]

**Supplementary figure 1.** pGlyco annotations of HAN^253^WTLTPLK (H5N4S2) (A) and HAN^253^WTLTPLK (H5N4S1) (B) of PON1 in the validation cohort.

**
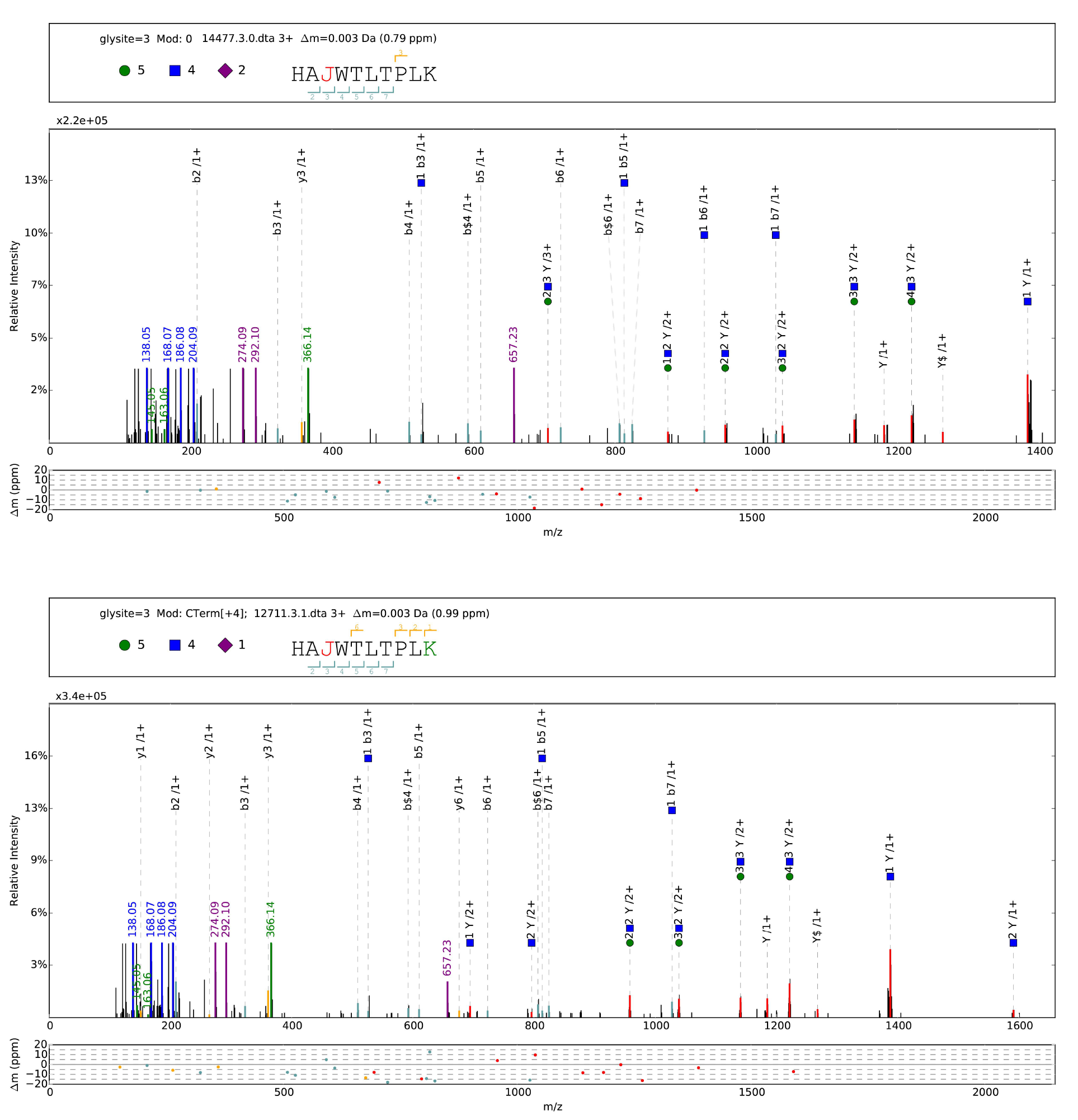
**

A

B
